# Transactions of the Medical and Physical Society of Calcutta. Vols. II. and III

**Published:** 1829-04-01

**Authors:** 


### VIII.

### TRANSACTIONS OF THE MEDICAL AND PHYSICAL SOCIETY OF CALCUTTA. Vols. II, and III. Calcutta. 1826-1827.

The gregarious propensities of mankind, the thirst for knowledge, and those social ties which are drawn tighter by distance from the "solum natale," have operated so strongly upon the minds of our professional brethren beyond the Ganges and Euphrates, that a medical society has not only been formed there, but volumes of transactions are elaborated with a fecundity and rapidity which form a singular contrast with the sterile and slow gestations (if we may use the expression) of certain ancient societies in the mother-country! We have not taken so much notice of these volumes, perhaps, as they deserved—chiefly, however, because a considerable proportion of their contents are of only local interest, which local interest will be sufficiently promoted by the Oriental circulation of the volumes themselves. It is our intention, nevertheless, to make our European and transatlantic readers acquainted with all those articles in the said volumes, which bear on the general scope of our science, and therefore shall proceed at once to work.

### ART. I.—ON DIARRHŒA HECTICA. By MR. J. TYTLER.

If pulmonary consumption carries off a great proportion of the European population, it would appear from Mr. Tytler, that a disease hitherto but little noticed, and never regularly described, commits equal ravages among the native inhabitants of India. Mr. Tytler has given it the name of "DIARRHŒA HECTICA," because, like its synonymous fever, "it seems to obtain habitual possession of the constitution, to operate upon it with scarcely any perceptible intermission, and, in general, to defy the most powerful remedies."

"It is perhaps not an exaggeration to say, that of the total deaths among the lower orders of the natives of Hindostan, three fourths are the effect of this disease, either idiopathic or as a terminating symptom. It is the scourge of crowded jails, and of hospitals filled with harrassed and fatigued Sipahees, and is the great avenue through which the swarms of naked and famished paupers and miserable infants that occupy the native bazars are continually vanishing." 1.

This disease is most common about the middle and end of the rainy season, and in the intensity of the cold weather. In the rainy season the transitions of temperature are very abrupt, and the effects of climate, Mr. T. observes, are greatly assisted by the quantities of raw and indigestible vegetables which the natives eat and give to their children. Another effect of this error in diet is the production of incredible quantities of round worms in the bowels—forty or fifty of which are sometimes voided at once—and occasionally two or three creep into the stomach and are discharged by vomiting. Mesenteric disease is also common. The common history of Diarrhœa Hectica is as follows :—

"A native, previously in apparent good health, is observed to appear languid, and unable to perform his usual labor. When questioned, he allows that he feels weak, but denies having any disease. On examination, his pulse is found feeble, and a little accelerated ; and at last he will state himself to have two watery stools in the course of the day, and two or three in the night. Shortly after this, his body begins to appear emaciated; the calves of his legs, particularly, fall away. The flesh, more especially about the wrists, has a peculiarly flabby and inelastic feel, as if the muscles had lost their tone, and the skin, like a loose bag, were too large to contain their diminished bulk: the cuticle peels off in patches, chiefly about the abdomen, fore-arms, and lower parts of the legs, so as to give the skin a faint mottled appearance. Collections of black sordes appear scattered up and down the body, which with a little care may be washed off, leaving the skin beneath of its usual color, so as to induce a belief that they are the consequences of the neglect of cleanliness, produced by the langour always accompanying the malady. Nothing particular is observable in the perspiration, except perhaps its being more scanty than usual. In the course of the disease, the pulse generally becomes more natural; though low and weak, it is slow and regular. The tongue also is quite clean, rather more so indeed than usual, and generally continues so all through the complaint; but the appetite begins to be impaired, the patient leaves half his common quantity of food uneaten, and complains that what he eats will not digest. He now confesses that '*pet chely*' he has a looseness ; that he has three or four stools in the course of the day, and as many in the night; on examining these, they are found to consist of a semi-liquid matter of two seemingly heterogeneous ingredients, one a pulpy mass approaching to a grey color, the other of bright yellow, intermixed with the former in streaks. To these are added occasionally, but not always, small quantities of blood. The stools are very copious, and have a peculiar odor, quite different from that of natural fæces, and indeed so distinctly their own, that I could almost engage to tell from the smell of an apartment, whether it contained a patient labouring under Diarrhœa Hectica. In this state matters go on for days and weeks, even sometimes for months ; the emaciation increasing, the appetite and strength diminishing, and the stools becoming more numerous, especially during the cold of the night. Then the feet, the hands, and at last the face, begin to become œdematous ; the abdomen sinks ; the eye loses its healthy lustre, the red vessels disappear from the conjunctiva, and it assumes that pearly whiteness so well known in phthisis, and long protracted disease of the liver. It is also frequently dimmed by a sordid exudation from the eyelids. Then the appetite totally disappears, scarcely any food is taken at all; and the patient, before too weak to walk, is now unable to sit up : he passes the greatest part of his time in a state of dozing, during which his eyes are always half open, probably from weakness of the sphincters ; the cornea is hid beneath the upper eyelid, and the white conjunctiva only appears. From this state of imperfect slumber, the patient, as long as his strength lasts, is perpetually roused to evacuate the contents of his irritable and restless bowels, which become more and more copious as his emaciated body can less and less afford them. At length the strength is too much diminished even for this, and the fæces come away involuntarily, and without intermission. The emaciation increases to a frightful degree, the superficial bones appear almost as distinct as in the skeleton, and the whole powers of life fail, except the incessant actions of the bowels, which seem impatient to hurry on the fatal event, and to labor as it approaches with increased irritability and vehemence. To close the narrative, the miserable patient, completely exhausted, expires in a flood of alvine discharge." 6.

In this deplorable condition, swarms of flies incessantly surround the patient, settle on his body, and greatly augment his distress. It is curious that, in certain cases, the disease assumes the *intermittent form*, the diarrhœa ceasing for two or three days at a time, and then regularly returning. Few dissections are, of course, permitted among the native Indians.

"Sometimes a partial redness, scarcely amounting to inflammation, is to be found in different parts of the small intestines. In some, the mesenteric glands are much enlarged, and apparently diseased; and this, upon the whole, is, I think, the most constant morbid appearance. In others the spleen is greatly increased in bulk, occasionally to a bulk much exceeding that of the liver, and of a very dense and compact structure. In one much emaciated female, a number of cretaceous substances, about the size of two peas each, were dispersed throughout its mass, and lodged in small cells, whilst the mesenteric glands were much enlarged. In an old man, the whole of the colon was completely stuffed with a white substance of a steatomatous appearance; the rectum was empty, and much inflamed. This patient was said to have been much addicted to the use of opium. But all these appearances are to be regarded as accidental accompaniments, and throw but little light on the real nature of the complaint." 9.

The remote causes have been already alluded to. The proximate cause, our author thinks, is, "an excessive and morbid excitement of the great intestines, by which they are perpetually disposed to evacuate their contents, mixed up with copious secretions from themselves, and, perhaps, from other parts of the intestinal canal." The methodus medendi may be comprised in a few lines. Opium, in some shape or other, is the sine qua non—all other remedies are of doubtful efficacy—and most of them prejudicial.

### II. CASES OF PHTHISIS PULMONALIS. By J. BIRD, Esq. A.M.

These cases are designed to illustrate the effect of climate, in varying the symptoms and changing the character of disease—and the author thinks that they tend to confirm the doctrine of Dr. Wilson Philip, regarding the causes and origin of one species of consumption. Phthisis is comparatively a rare disease in India, and is seldom produced there but when a strong predisposition exists in the constitution.

"But when the action which causes tubercles of the lungs has been once excited, the progress of it is very rapid, and, as far as I know, always fatal. Whatever advantage may be expected from a warmer atmosphere, and change of climate, by those suffering from the disease, is more than counterbalanced by the disposition shewn in the system to dyspeptic and hepatic complaints, which follow as the natural consequences of the great increase of heat to which the body becomes exposed, on leaving an extratropical for a tropical climate." 191.

After speaking of the excitement produced in the system by the high range of atmospheric temperature between the tropics, our author proceeds thus :—

"This state is soon followed by a diminished inclination for food; by fulness at the epigastrium; pain in the loins; flatulency; thirst; a foul state of the tongue; constipated bowels; and, in short, by all those symptoms which indicate increasing derangement of the digestive organs. The large intestines begin, at length, to sympathise with the increasing torpor of the stomach and skin. The patient complains of pain in the site of the colon; has occasional watery purging; or is affected by dysenteric irritability of the bowels, and passes mucus. During the attacks, his cough is much aggravated, in consequence of the increased difficulty he meets with in bringing up the mucus secreted in the lungs. He has now constant pain and tenderness within the ribs of the right, or left side; symptoms which may be readily mistaken for those of hepatitis, or splenitis. He also becomes daily more emaciated; and suffers from alternate profuse diarrhœa, and hectic sweating; which are soon followed by increase of cough and purulent expectoration. At last, death comes, to terminate his misery." 192.

During eight years' practice among European soldiers, our author has met with many of these cases—but none where recovery took place. Some cases are related in illustration, but which we shall pass over. Mr. Bird makes some observations on the *diagnosis*. He is evidently unacquainted, or at least inexperienced, in the modern diagnostic indications afforded by the stethoscope, and, therefore, his observations on this particular point are comparatively devoid of interest, in our present advanced state of knowledge. The following extract contains much sound advice.

"In regard to the treatment of phthisis, so far advanced, as that inflammation has been excited in the lungs by the tubercles, I cannot venture to recommend any particular mode; since every thing usually employed on such occasions has failed, in my hands, of arresting the disease in its fatal career. I am, therefore disposed to think we should place our sole reliance on those remedies which are best calculated to correct that derangement of the general health preceding this inflammatory stage; and which, by restoring the tone of the digestive organs, and renewing the healthy action of the liver and skin, are the only sure preventives of the injurious effects of changes of temperature, or other external agents. Flannel worn next the skin, is the first, if not the most useful in the list of these preventives; and, as has been shewn by experience, it is the best and most powerful safeguard against all the diseases incident to tropical climates. In order to increase the secretions of the liver, five or ten grains of blue-pill should be given at bedtime, and the nitro-muriatic acid foot-bath used at the same period. When the bowels are torpid, a little rhubarb, with a double quantity of magnesia, taken two hours before dinner, will effectually correct this state, until their natural action returns; and this will be much assisted by half an hour's rubbing of the skin, either by a towel or flesh-brush, previous to the patient's dressing himself in the morning. Along with these, exercise on horseback, temperance, and moderation in eating, are measures not to be neglected, as they contribute much to the improvement of the general health. By a practice such as these, I have had the satisfaction to see the constitution rally, in cases where nothing but a change of climate gave any hope of success; and to such I should trust for the cure of that dyspeptic state which precedes and accompanies phthiscal affections within the tropics. Muriated tincture of iron may be also usefully employed along with this, when the circulation is languid; but, as it is apt to produce constipation, it should be given in small doses of ten or twelve drops; and, after being used two or three days, should be left off, and again had recourse to. I have prescribed it with advantage in several dyspeptic cases, and the other preparations of this mineral deserve a further trial, particularly for that dyspepsia preceding phthisis." 206.

### ART. III.—ON THE TREATMENT OF INDIAN RHEUMATISM. By J. HENDERSON, Esq.

Mr. Henderson has published a paper on the above subject in the Edinburgh Medico-Chirurgical Transactions, which has been noticed in a former Number of this Journal. The following extract contains some curious practical observations on the chronic state, stage, or rather sequelæ, of rheumatism, which may possibly afford some useful hints to the European practitioner.

"The disease generally comes on suddenly, attended with violent pyrexial symptoms, which last a shorter or longer time ; and the longer they continue, and the more violent they are, the secondary symptoms are more urgent, and are removed with greater difficulty. At first, the evident and only effectual treatment is antiphlogistic: the lancet is to be used with no sparing hand, and the bleeding has often to be repeated five or six times, within the first five days. Absolute rest, purgatives, and blisters, are the remedies which appear next best suited to the first stage: but the last must be applied with caution, because they are unsuited to the second stage, and their healing materially interferes with more effectual applications. Diaphoretics appear to be of little or no use in this stage of the disorder.

"The pulse and tongue have indicated a great diminution in the inflammatory symptoms, the former being soft, small, and quick, and the latter glassy generally, and white, we must resort to a new plan of treatment. The remedies I have found most successful are mechanical frictions and motions of the joints, champooing, and exercise. The last, however, is the principal, and perhaps the only one that can be implicitly relied on. It is by no means sufficient that the patient should extend the limbs as much as the pain he suffers or the stiffness will admit of; neither is it sufficient that this operation be performed by an assistant. The operation must be done with comparative violence, and so as to produce active perspiration, or it is useless. The riding in a hackery driven with violence, or on horseback, the horse being at the hard trot, or on a camel at its roughest pace, will be found the most effectual remedies we are possessed of. The agony or cries of the patient, which are diminished each time the remedy is applied, must not divert the surgeon from persisting in it. He must submit to it twice, and when the season will admit of it, three times in a day ; but of course, at first, it should be continued for a very short period at a time. The patient must, on alighting, be well shampooed, his joints rubbed with violence, so as however not to injure the external skin, and he is then to be allowed to sleep under a thick covering to encourage perspiration. Gentle purgatives are evidently required during the cure, as the whole constitution is then suffering from a species of hectic. The appearance of the tongue and change in the pulse are the best indications to the surgeon of the progress of the cure. By perseverance in this plan, I have cured many who have come under my care, several months after the commencement of the disease, and relieved many whose joints had become rigid, and where disorganization had ensued. From the perusal of the above, the reason will become obvious why rheumatic affections occurring on a march, are so severe and inveterate, as well as the cause, why it so frequently occurs, that sepoys allowed in this disorder to proceed to their homes by the surgeon, after having exhausted his remedies, return in a short period perfectly cured. In the first place, the sepoy is carried about, often on a hackery, at a period when rest is most urgently required, by which the inflammatory state of the joints is in many cases so much increased, as to cause rapid disorganization. In the second case, the sepoy returning to his home at a distance, finding it impossible to walk, gets on either by means of a hackery or *tattoo,* which produces all the effects the Surgeon has in vain been endeavouring to produce. The subject is of some importance to the Government; for from what I have seen I am led to believe, that one half of the men invalided annually, are rheumatic cases, a great proportion of which have been commenced on the line of march." 429.

### ART. IV.—ON SLOUGHING ULCERS. By J. LANGSTAFF, ESQ. Benares.

The following extract will shew the influence of malaria on the general health, and through that, on local diseases.

"Intractable ulcers, of an erysipelatous and sloughing character, have long been numerous in the hospitals at this station, as well as at others, even in men who never were to the eastward: this fact, after the liability to invasion of Cholera, in the epidemic form, amongst the Natives, is the most remarkable feature in the medical history of the times I know of. Two patients at this station, borne on the present return, died under the circumstances in question; one of sloughing of the genitals, the other of the kind of ulcer above-mentioned, in his leg. With reference to which case (the latter) Mr. Row, of the 5th Extra Regiment, has afforded the following remark: 'Ulcers have been very prevalent in the 9th Regiment for some time past. Many men have been admitted with common boils, which on being laid open, or bursting spontaneously, immediately degenerated into phagedenic ulcers; others again have been admitted with such description of sores from the lines, and from leave, or from command. They are very obstinate, and are with difficulty affected or altered in their character by any mode of treatment: such was the case of ulcer that proved fatal. The sore several times put on a healthy aspect, and as often degenerated, until at last the constitution sunk under its influence. The cause of this disposition to phagedena I am not able to discover, but suppose it to depend on a peculiar state of the atmosphere. I am informed that this state has existed in other regiments at this station, equally with the 5th Extra, for above a twelvemonth.' If I might add any thing to the above remark, I would say, from my own observation, that the habit of the patient in question was so unhealthy and irritable, and he became so much reduced before the hope of cure was relinquished, that amputating (a precarious recourse at any period in such a state of the system) ultimately could not be ventured on. The returns from the other stations of native troops, afforded little to enlarge on. Considerable healthfulness on the whole prevails, excepting in regard to the Local corps at Goruckpore, which has, at various periods of late, been very unhealthy. Assistant Surgeon Mr. Thomson, in charge of the above corps, has added the following remark to his monthly return. 'A very large proportion of the cases of fever which have been admitted during this month, are of the intermittent type; and in those cases in which the patient had not been much reduced by the effects of former sickness, the fever has generally proved rather mild, although very apt to return after a considerable intermission. But in those cases where the patients were very weak from the effects of last year's sickness, it has shewn itself peculiarly obstinate. The means which have proved most effectual have been calomel in small doses, united with diaphoretics. Arsenical solution was given largely, but I think the preference was to the former. Bark was used in several cases, but apparently without benefit. Where a more plethoric habit of body was observable, after one or two brisk purgatives, the blue-pill was found decidedly beneficial. As the two fatal cases occurred before I joined, I have no remarks to offer.'" 420.

[To be continued.]

